# Three-dimensional imaging of xylem at cell wall level through near field nano holotomography

**DOI:** 10.1038/s41598-021-83885-8

**Published:** 2021-02-25

**Authors:** Tim Koddenberg, Imke Greving, Johannes Hagemann, Silja Flenner, Andreas Krause, Daniel Laipple, Kim C. Klein, Uwe Schmitt, Max Schuster, Andreas Wolf, Maria Seifert, Veronika Ludwig, Stefan Funk, Holger Militz, Martin Nopens

**Affiliations:** 1 Wood Biology and Wood Products, Faculty of Forest Sciences and Forest Ecology, University of Goettingen, Büsgenweg 4, 37077 Göttingen, Germany; 2 Institute of Materials Physics, Helmholtz-Zentrum Geesthacht, Max Plank Straße1, 21502 Geesthacht, Germany; 3 Deutsches Elektronen Synchrotron-DESY, Notkestrasse 85, 22607 Hamburg, Germany; 4 Thünen-Institute of Wood Research, Leuschnerstraße 91, Hamburg-Bergedorf, 21031 Hamburg, Germany; 5 Erlangen Centre for Astroparticle Physics, Friedrich-Alexander University Erlangen-Nürnberg (FAU), Erwin-Rommel-Strasse 1, 91058 Erlangen, Germany

**Keywords:** Biomaterials, Imaging techniques, Structural biology

## Abstract

Detailed imaging of the three-dimensionally complex architecture of xylary plants is important for studying biological and mechanical functions of woody plants. Apart from common two-dimensional microscopy, X-ray micro-computed tomography has been established as a three-dimensional (3D) imaging method for studying the hydraulic function of wooden plants. However, this X-ray imaging method can barely reach the resolution needed to see the minute structures (e.g. pit membrane). To complement the xylem structure with 3D views at the nanoscale level, X-ray near-field nano-holotomography (NFH) was applied to analyze the wood species *Pinus sylvestris* and *Fagus sylvatica*. The demanded small specimens required focused ion beam (FIB) application. The FIB milling, however, influenced the image quality through gallium implantation on the cell-wall surfaces. The measurements indicated that NFH is appropriate for imaging wood at nanometric resolution. With a 26 nm voxel pitch, the structure of the cell-wall surface in *Pinus sylvestris* could be visualized in genuine detail. In wood of *Fagus sylvatica*, the structure of a pit pair, including the pit membrane, between two neighboring fibrous cells could be traced tomographically.

## Introduction

In plant research, imaging methods are crucial to understand and characterize the mechanical and physiological functions of plants (e.g. hydraulic conductivity). Thereby, the three-dimensionally very heterogeneous and microscopic structures of plants, especially wooden plants, call for imaging methods with sufficiently high magnification to examine anatomical structures. Since the development of the microscope in the seventeenth century, magnification has been put to its limits to unveil what has been unseen so far^1^. Pioneering microscopy tools have been evolved into a broad array of techniques (e.g. light, electron, and atomic-force microscopy) allowing multi-modal analyses paired with high magnifications^1,2^. For instance, electron microscopy techniques have been applied revealing and quantifying the influence of the shape of pit pores and the pit membranes^3–5^. However, most of the common imaging techniques are based on two-dimensional (2D) sections, and thus often lack three-dimensional (3D) information.

In the last decades, X-ray micro-computed tomography (microCT) with resolutions down to 1 µm has been established in many domains of xylary plant research^6–9^. Despite the confined resolution, microCT combines, unlike most common microscopy methods (e.g. light and electron microscopy), the possibility of non-destructive 3D examinations of the internal plant structure. This advantage has often been used for studies on the hydraulic architecture of trees and other xylary plants^10–14^ representing one of the major trends in the field of tree-water relations since a few decades^15–18^. Several anatomical traits like tracheid diameter in softwoods and vessel size in hardwoods play a role in determining the trade-off between hydraulic efficiency and hydraulic safety, whereby pits with their membranes are believed to represent the key structure in this complex system^19,20^. Despite the minute pit size, pits have been three-dimensionally examined using microCT. For instance, Trtik et al.^21^ provided morphological visualization of bordered pits in *Picea abies* in 3D using synchrotron radiation, while Koddenberg et al.^22^ used a laboratory microCT to evaluate the volumetric dimension of bordered pits in *Pinus sylvestris*. However, microCT can barely reach the resolution needed to visualize the pit membrane. Pit membranes may vary distinctly in their thickness from 100 nm up to over 1 µm, whereby the majority has a thickness of below 500 nm, especially intervessel pits in angiosperms^18,23^.

Apart from microCT, X-ray nano-tomography (nanoCT) technologies have emerged in the last years as an up-coming tool for studying organic structures at the nanoscale level^20,24^. Using ptychographic X-ray computed tomography, Kaack et al.^20^ pioneered remarkable 3D reconstructions of intervessel pits, including the pit membrane, in *Cinnamomum camphora*. With regard to the nanoscale imaging of wood, it is important to mention that also other tomography methods exist (e.g. using serial electron microscopy images^2,25^), which can be superior to X-ray tomography methods when it comes to detail recognition. Methods like scanning electron microscope focused ion beam (SEM–FIB) tomography however have disadvantages like e.g.: Being a destructive technique, having a non-isotropic spatial resolution and the specimens have to be imaged under vacuum conditions. In addition, scan times in SEM–FIB tomography, like laboratory microCT and ptychographic tomography^20^ can exceed several hours. In contrast, synchrotron beamlines with setups for full-field micro and/or nano-tomographic imaging offer acquisition times of less than one hour. On the one hand the tomographic scan time depends on the properties of the instrument (e.g. photon flux, detector deadtimes, exposure time, numbers of steps in the rotation) on the other hand the nature of the specimen itself, like its composition and thickness play an important role. As wood, or in general biological specimens, are composed of light and thus weakly absorbing elements, a phase sensitive imaging method is needed. Magnified inline near-field holography (NFH) is such a phase sensitive full-field imaging technique^26,27^. NFH is readily implemented by placing a sample in the diverging beam behind focusing optics^28,29^. The holographic projections at the detector plane are then formed by free-space propagation of the X-rays, therefore NFH is a lens-less imaging technique. The actual image is formed *a-posteriori* by phase retrieval on the intensity-only measurements^30^.

In this study, we applied NFH for the first time to wood as a valuable and dose efficient method to image biological tissues at cell-wall level with nanometric resolution in reasonable time at ambient conditions. As biological tissue, we used xylary tissue material of *Fagus sylvatica* and *Pinus sylvestris*. Combining method and material, the nanometric resolution of the NFH tomography enabled us to visualize the pit in three dimensions with an effective pixel pitch of 26 nm.

## Materials and methods

### Material preparation

**Figure 1:**
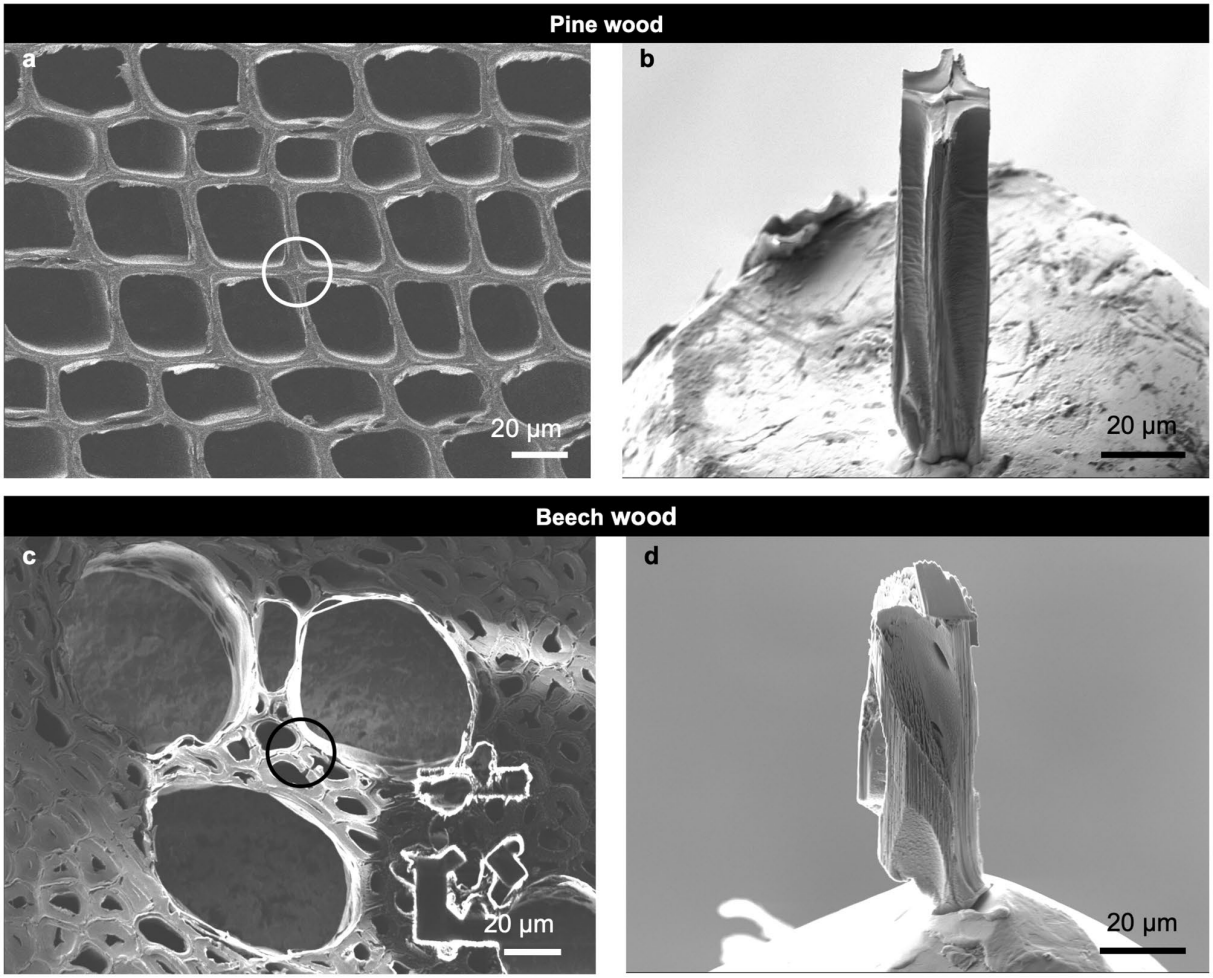
Wood specimens before (left) and after (right) FIB preparation, respectively. The location of the prepared specimens (**b**,**d**) within the wood tissue is highlighted by circles in (**a**) and (**c**). (**a**,**b**) Pine earlywood. (**c**,**d**) Beech earlywood. Scale bars: 20 µm.

The current study utilized a wood material commonly used for technological applications (e.g. building materials). More precisely, earlywood from a tree-ring located in the sapwood of the angiosperm species *Fagus sylvatica* L. (beech) and the conifer species *Pinus sylvestris* L. (pine) were used. During the sample-preparation process, each specimen has been dried totally (due to the SEM handling in vacuum). The ambient moisture condition during scanning was supplied by the moisture humidity generator MHG 100 ProUmid, Germany. Due to the requirements of the experimental setup, small specimen dimensions were demanded. At first 50 µm thick transverse sections were prepared with a sliding microtome. The specimen dimensions were approximately 40 × 40 × 90 µm^3^ (Fig. 1a–d). For specimen preparation, a scanning electron microscope (SEM) Auriga from Zeiss equipped with the focused ion beam (FIB) column Canion was used. The FIB milling was performed on the wood using gallium (Ga) ions with currents of mainly 10 nA to excavate the desired wood structure. Subsequently, the specimens were mounted on the tip of a metal pin using a micro-manipulator and a deposition method provided by the implemented gas injection system.

### Experimental setup

The measurements were performed at the nano-tomography setup at the imaging beamline P05 at PETRA III, operated by the Helmholtz Zentrum Geesthacht^31^. The instrument is designed for full-field transmission X-ray microscopy as well as holotomography methods. A double crystal monochromator was used at 11 keV for illuminating a Fresnel zone plate (FZP) of 300 µm diameter with an outermost zone width of 50 nm^32^. A beam stop was implemented covering 60% of the FZP, blocking the primary (unfocused) beam. In the focal distance of the FZP order sorting apertures were positioned to suppress higher diffraction orders of the FZP. The achievable theoretical focus size of this setup is 83 nm. For the holotomography experiment the samples were placed on the high precision rotation axis, and tomography scans performing a 180-degree rotation were recorded at three different defocus positions (Table 1). A Hamamatsu camera (C12849-101U) with a 10 μm thick Gadox scintillator directly coupled to the sCMOS chip allows imaging at high photon efficiency. Due to the unique geometry of the P05 beamline, the camera could be placed at a distance of 16.269 m behind the focus (Table 1) to allow for an isotropic effective pixel size of 26 nm in the resulting images. Given the point spread function of the detector and taking the geometrical limitations into account, the spatial resolution is expected to be in the order of 150 nm.

**Table 1:** Scanning parameters for near-field nano-holotomography specimens.

Photo energy (keV)	11.0
Exposure time (s)	1
Number of projections	900
Angle step size (°)	0.2
Distance to detector (m)	16.269
Defocus distances (mm)	[70, 71, 72]
Effective pixel size (nm)	26
Field of view (µm)	53.2

### Data processing

**Figure 2:**
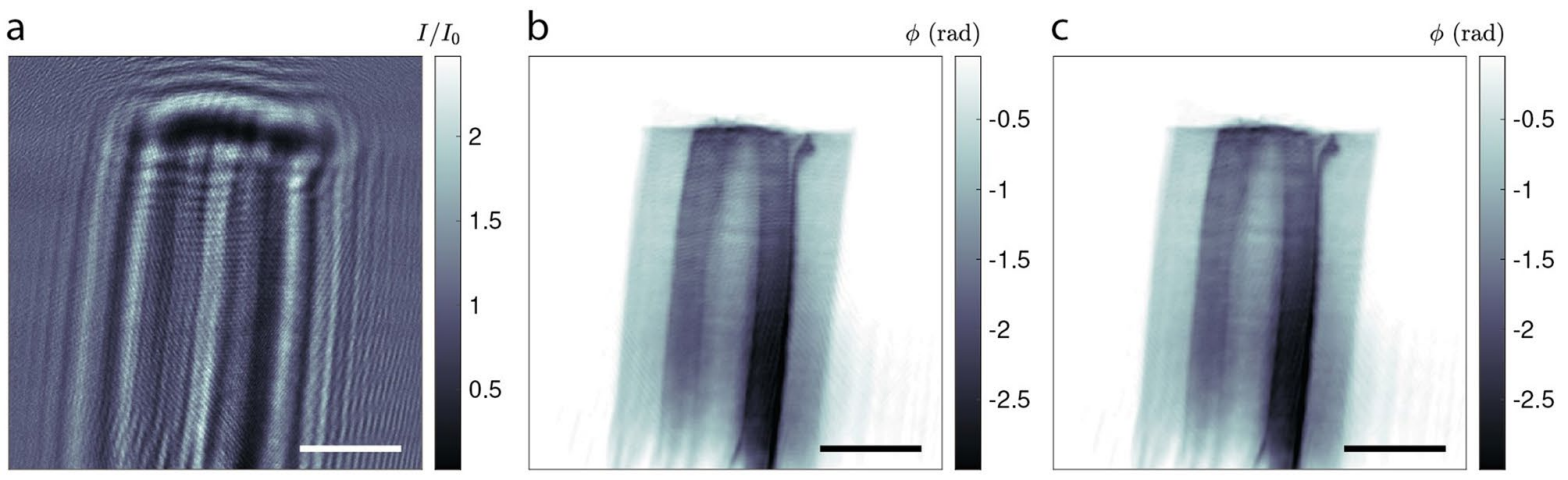
NFH measurement of the pine specimen. (**a**) Flat-field corrected NFH image (*I*/*I*_0_) at a Fresnel number of *F* = 10^−4^. (**b**) The recovered phases (ϕ (radian)) of the specimen. The stripe artifacts from the illumination leave an imprint in the reconstruction. (**c**) The result of a morphological component analysis on (**b**) to remove the artifacts. Scale bars: 10 µm.

The data processing involves three stages: (i) preparing the data for phase retrieval, (ii) the phase-retrieval for all projections, and (iii) the tomographic reconstruction. The initial data preparation consists of flat-field correcting the projections and calculation and refinement of the geometry parameters, (*i.e.* magnification and Fresnel number ($F$)). Figure 2a shows the corrected hologram of the sample. The phase reconstruction in Fig. 2b is computed from three holograms recorded at defocus distances of 70 mm, 71 mm, and 72 mm. The holograms are then used in an iterative projection based algorithm^30^. In the algorithm certain support and range constraints need to be applied in order to reconstruct all 900 projection angles. In the following, the filtered projections have been aligned and then used as an input for the tomographic reconstruction. The reconstruction of the slices was performed using the gridrec algorithm of the open-source Python package TomoPy used for tomographic data processing and image reconstruction^33^.

### Data analysis

After data processing, the image visualization was performed on stacks of 2D cross-sectional slices utilizing the Avizo software package (FEI, Thermo Fisher Scientific, Hillsboro, Oregon, USA). The visualization was accomplished on selectively chosen regions of interest (ROIs). The segmentation required for 3D visualization was accomplished on ROIs using the region-growing based segmentation algorithm in Avizo. This segmentation technique compares a seed voxel with other voxels of the ROI, based on two criteria: spatial proximity and similarity in grayscale values. The isotropic voxel size of the 3D reconstructions was 26 × 26 × 26 nm^3^. After segmentation, the visualization was realized with the visualization tools in Avizo. Apart from visualizations, quantifications were performed by measurements in 2D and 3D. The measurement of the pit volume was achieved by counting the voxels of interest.

## Results and discussion

**Figure 3:**
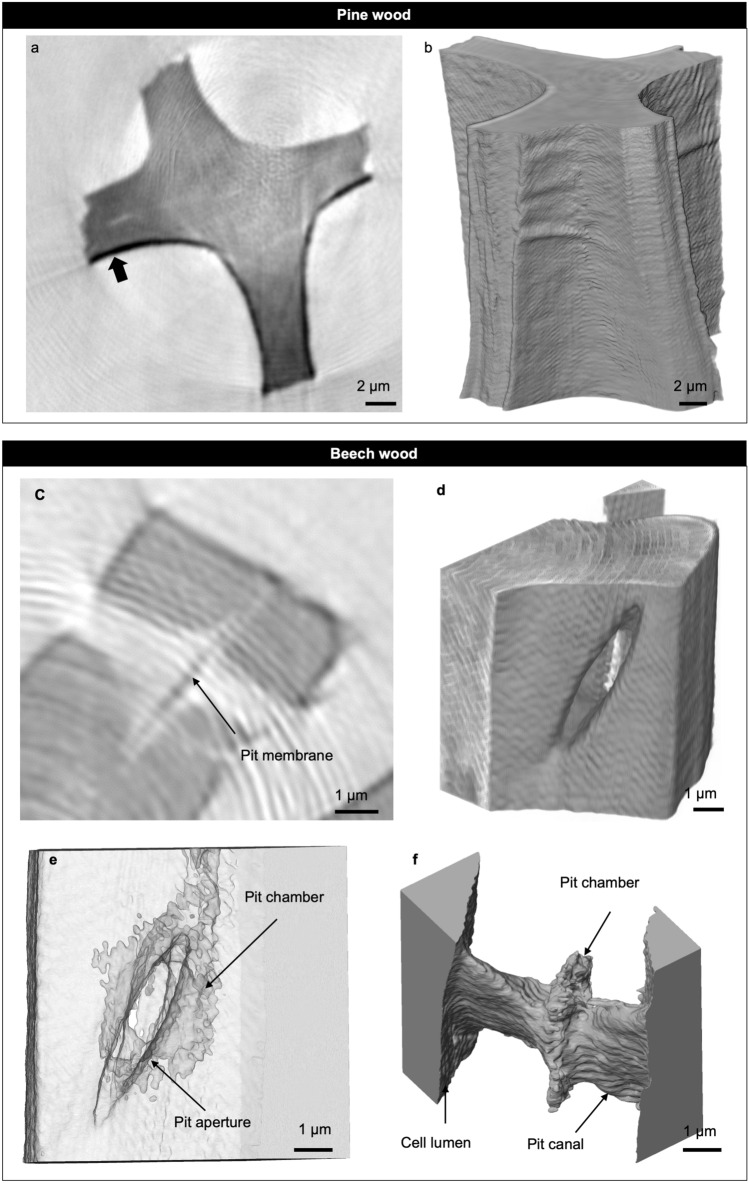
Tomographic imaging of pine and beech earlywood in a dry state. (**a**,**b**) Cell-wall between tracheids in pine. Scale bars: 2 µm. (**a**) Cross-sectional slice of the sample revealing gallium coating on the cell-wall surface (arrow). (**b**) Volume rendering of the cell wall. (**c**–**f**) Tomographic visualization of a pit pair between fibrous cells in beech. Scale bars: 1 µm. (**c**) Two-dimensional slice of the ROI. The arrow indicates the pit membrane. (**d**) Volume rendering of the cell wall revealing the one side’s aperture of the pit. (**e**) Transparency cell-wall view reveals the pit chamber in the pit’s center. (**f**) Three-dimensional void visualization of a pit pair connecting the lumina of two neighboring fibrous cells.

We first evaluated the NFH data/images of pine and beech specimens in 2D and 3D in order to evaluate if our specimen preparation and the chosen NFH setup at the beamline were appropriate for the imaging of wood at nanometric resolution. The phase retrieval algorithm works best for homogenous specimens^30,34^, ideally pure phase objects. In this study, the heterogeneous specimens consist, apart from air, of two components (wood and Ga) with very different properties regarding their refractive index including X-ray attenuation and phase shift. In the grayscale images, local Ga implantations on the surface of the specimens caused by FIB application were observed (Fig. 3a). It should be noted that the use of Ga was inevitably during FIB milling. Ga was identified by the darker voxel shades indicated in Fig. 3a regarding their higher index of refraction compared to the organic wood tissue. This Ga implantation, though, posed a challenge for quantitative phase retrieval. In order to circumvent this issue, the tomograms were recorded at three defocus positions, which pose as additional constrains in the phase retrieval process. Apart from Ga implantation, the recovered projections showed an imprint of a ripple pattern (Fig. 3a,c). This pattern originates from artefacts in the illumination and was mitigated by applying a morphological filter^35^ on the projections (Fig. 2c). However, the 2D and 3D reconstructions in Fig. 3c,f reveal that the ripples could not be removed completely. Given that the spatial period of the ripples is considerably larger than the length scale and orientation of the features studied here, the structures could still be resolved in detail.

In the segmentation process, these ripple patterns could not be removed. The Ga contamination however, clearly observed by the darker voxels could easily be removed by choosing the right threshold. Therefore, we achieved detailed visualizations of the cell walls of exemplary pine and beech wood. The achieved voxel size allows unique and non-destructive 3D insights of beech and pine wood at nanometric resolution and under ambient conditions. Kaack et al.^20^ demonstrated remarkable illustrations of the cell wall in *Cinnamomum camphora* also at high spatial resolution but under cryo-conditions using ptychography.

Figure 3b shows that NFH is a valuable tool for imaging the cell-wall surface compared to SEM imaging in Fig. 1b, respectively. Measurements of the pure double wall of the four outer segments (Fig. 3a) revealed an average wall thickness of 3.617 µm. That is in good agreement with values for *P. sylvestris* reported in the literature^36,37^.

Aside from the imaging of the cell-wall surface, image analysis of NFH data also enables enhanced views of the internal xylem structures. Imaging of air-filled regions (= voids) within the wood tissue makes it possible to detect a pit pair located between two neighboring fibrous cells in *F. sylvatica* (Fig. 3c–f). The pit morphology itself has been three-dimensionally rendered by microCT^21,22^ at low spatial resolution and by nanometric ptychographic tomography at cryogenic conditions^20^. The pit canal and pit cavity could also be visualized using NFH. The tomographic reconstruction reveals the slit-like shape of the pit canal clearly visible (Fig. 3f). As expected, nanoCT is in clear superior to microCT when it comes to the detection of the unaspirated pit membrane in the center of the pit chamber (Fig. 3c). Nonetheless, it was not possible to separate the pit membrane properly in 3D, likely because of the presence of the ripple pattern mentioned above. The fact that dry wood was used might additionally challenge the separation of the pit membrane because the thickness of pit membranes becomes distinctly reduced during dehydration as shown especially for intervessel pit membranes (e.g. Li et al.^38^, Zhang et al.^39^).

Also, the dataset enabled determining common pit dimensions. The pit shown in Fig. 3c–f has a maximal chamber diameter of 4,230 nm, and a pit-membrane thickness at the center of 428 nm as obtained using 2D slices. Furthermore, a width of 1.053 µm was measured as a maximum length of the slit-like pit canal. Both values, pit-membrane thickness and pit-chamber diameter, are in general agreement with literature values of pits^40,41^; although mostly bordered pits were measured^42^ in the intervessel walls. However, it should be mentioned that a clear differentiation between fiber tracheids and libriform fibers within the morphological continuum by the size of a single pit appears difficult. In addition to these common pit measurements, we quantified the pit-void volume of 21.44 µm^3^ (considering a voxel count of 1,220,270 and an isotropic voxel size of 26 nm). The volume includes the pit canal and the pit chamber. Comparing this value with intertracheid bordered pits in *P. sylvestris* analyzed by microCT^22^ exemplifies that the pit volume of this particular pit in *F. sylvatica* is 42 times smaller than the volume of earlywood pits and still 16 times smaller than the latewood pits in *P. sylvestris*.

Despite the detailed imaging of the wood structures at cell-wall level, NFH, as every imaging method, comes with some limitations. We already mentioned the tomography artifacts emerged upon NFH leading to decrease in image quality. These artefacts however are not due to the technique but due to instrumental limitations (structures on the monochromator). Another valuable method for nanometric imaging is ptychographic X-ray tomography that enables nanoscale resolution combined with scanning under cryogenic condition^20^. Scan times however do exceed several hours, in contrast to the presented NFH method, resulting in a higher dose which is a critical factor in biological samples. A common limitation of scanning and full-field methods at nanometric resolution is the need of miniature specimen dimensions. Hence, there is a trade-off between the field of view (FOV)—corresponding to the physical volume scanned—and the resolution intended. Therefore, unlike in microCT^7,8^ specimens have to be in the size range of 10th of µm (Table 1) and need to be prepared using FIB milling. This can induce artefacts due to the Ga layer. An advantage of NFH over other full-field methods, like transmission X-ray microscopy is, that the FOV and hence the resolution, can be freely scaled by the focus to sample distance. Also, ROI scans of larger specimen are possible. Thanks to the relatively short scan times and the high flexibility of the presented method the implementation of an in situ environmental cell for imaging pit structures at different humidity levels is planned. Overall NFH demonstrated its value for anatomical examination of xylem properties in this study.
